# Laparoscopic Segmental Duodenectomy With Antrectomy as a Feasible Strategy for Proximal Non-ampullary Duodenal Cancer in Octogenarians: A Report of Two Cases

**DOI:** 10.7759/cureus.111223

**Published:** 2026-06-21

**Authors:** Osamu Inamoto, Shuhei Sakamoto, Takashi Fujimoto, Kazuhiro Nishiyama, Ryotaro Ogawa, Izumi Komoto

**Affiliations:** 1 Department of Surgery, Kansai Electric Power Hospital, Osaka, JPN

**Keywords:** antrectomy, function-preserving surgery, laparoscopic surgery, non-ampullary duodenal cancer, older adults, segmental duodenectomy

## Abstract

The optimal therapeutic approach for early-stage non-ampullary duodenal cancer (NADC) remains challenging, particularly for tumors that are difficult to manage with endoscopic resection (ER) but do not strictly require highly invasive pancreaticoduodenectomy (PD). Here, we report two octogenarians with early-stage proximal NADC successfully treated with laparoscopic segmental duodenectomy with antrectomy (LSDA), a procedure that provides oncological radicality while minimizing invasiveness. An 89-year-old woman presented with a 30 mm Borrmann type 1-like tumor extending from the duodenal bulb to the pyloric ring, for which ER or laparoscopy-endoscopy cooperative surgery (LECS) was considered high risk due to tumor location and size. An 85-year-old woman had a tumor with suspected submucosal invasion (T1b) at the superior duodenal angle, and although no lymph node metastasis was evident, PD was considered excessively invasive given her age and frailty. In both cases, LSDA with selective lymph node dissection was safely performed, followed by Billroth II reconstruction. Both patients achieved R0 resection and had uneventful postoperative courses, with discharge within 14 days, and they remain recurrence-free at 58 and 35 months, respectively, with preserved nutritional status and quality of life. LSDA is a function-preserving and oncologically sound treatment option for older adults with early-stage NADC, serving as a versatile approach that bridges the gap between endoscopic treatment and PD.

## Introduction

Non-ampullary duodenal cancer (NADC) is a rare gastrointestinal malignancy [1]. Although the Clinical Practice Guidelines for Duodenal Cancer (2021) aimed to standardize treatment strategies [2], the optimal surgical approach remains controversial, particularly in balancing oncological radicality with minimal invasiveness.

Management of NADC ranges from endoscopic resection (ER) [3] and laparoscopy-endoscopy cooperative surgery (LECS) [4] for superficial lesions to pancreaticoduodenectomy (PD) for advanced disease [5]. However, a clinical gap exists for intermediate cases, such as superficial tumors >20 mm or those with suspected submucosal (pT1b) invasion [2]. In such cases, ER or LECS may carry risks of incomplete resection or postoperative stenosis, particularly near the pylorus, whereas PD may be excessively invasive, particularly in octogenarians, where life-threatening complications can be prohibitive [6].

Current guidelines suggest that limited duodenal resection with regional lymphadenectomy may be considered for selected patients, based on tumor- and patient-related factors [2]. This highlights the need for a function-preserving strategy that bridges the gap between local resection and PD.

In this report, two octogenarians with early-stage proximal NADC were treated with laparoscopic segmental duodenectomy with antrectomy (LSDA). We demonstrate that LSDA achieved adequate resection margins and lymph node dissection while minimizing surgical stress, supporting its role as a function-preserving and oncologically sound approach.

## Case presentation

In this case series, performance status was graded using the Eastern Cooperative Oncology Group Performance Status (ECOG-PS) [7], and frailty was classified according to the Clinical Frailty Scale (CFS) [8].

Case 1

An 89-year-old woman (ECOG-PS 2, CFS 6) presented with melena. Upper gastrointestinal endoscopy revealed a 30 mm superficially elevated tumor in the duodenal bulb extending to the pyloric ring (Figure 1a). Biopsy and contrast-enhanced computed tomography (CT) suggested an early-stage lesion without pancreatic invasion (Figure 2a), although the tumor’s size and location posed therapeutic challenges.

**Figure 1:**
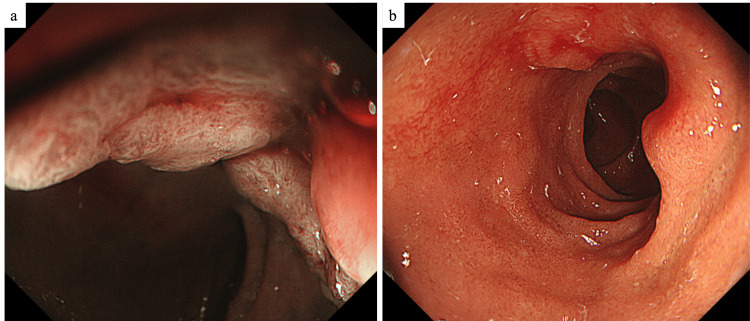
Endoscopic findings (a) Case 1. A bulky elevated lesion extending to the pyloric ring is visible in the duodenal bulb. (b) Case 2. A type-0-Is or small type-2 tumor located at the superior duodenal angle, suggesting possible deep submucosal invasion.

**Figure 2:**
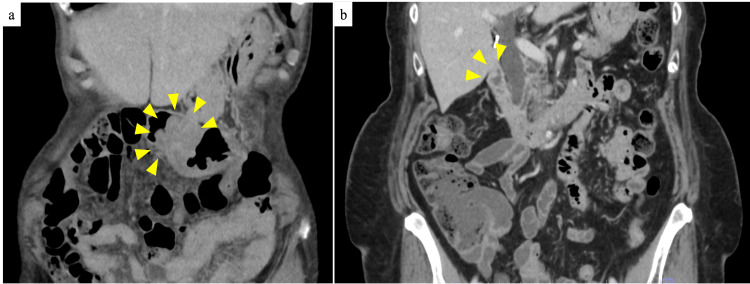
Preoperative computed tomography (a) Case 1. Tumor (arrowhead) appearing as a mass localized within the duodenal wall; however, the precise depth of invasion is difficult to determine. No apparent pancreatic invasion is observed. (b) Case 2. Although the tumor itself appears faint on computed tomography, its location is identifiable (arrowhead), with no signs of advanced disease or lymph node metastasis.

ER was deemed unsuitable due to the tumor’s characteristics, particularly its proximity to the pylorus, which increased the risk of postoperative stenosis and difficulty securing a clear horizontal margin. LECS was also considered suboptimal because it would require complex reconstruction near the pylorus. Therefore, LSDA was selected to achieve R0 resection while preserving gastrointestinal continuity.

Case 2

An 85-year-old woman (ECOG-PS 2, CFS 5) was referred for evaluation of a tumor at the superior duodenal angle (Figure 1b). Endoscopic-guided ultrasound (EUS) findings suggested deep submucosal invasion (type 0-Is or small type 2), and contrast-enhanced CT confirmed the tumor location without evidence of lymph node metastasis (Figure 2b). Preoperative MRCP demonstrated normal pancreatic duct anatomy, confirming that the minor papilla would not interfere with the resection.

Although lymph node metastasis was not detected on imaging, ER was considered insufficient due to the risk of regional lymph node metastasis associated with suspected deep submucosal (T1b) invasion. Furthermore, PD was considered excessively invasive given her age and frailty. Therefore, LSDA with selective lymph node dissection was performed to achieve a balance between oncological radicality and minimal invasiveness.

Surgical procedure (common to both cases)

Both patients underwent laparoscopic surgery using a five-port technique in the split-leg position. The operative schema (representing Case 2) is shown in Figure 3. After opening the lesser sac, we performed dissection of lymph node station 6 (infrapyloric nodes), and then divided the right gastroepiploic vein and artery (Figure 4a). The supraduodenal artery was then divided to circumferentially encircle the duodenum. Next, we carefully detached the duodenum from the pancreatic head by dissecting the peripancreatic connective tissue to ensure full mobilization for the subsequent segmental resection (Figure 4b).

**Figure 3:**
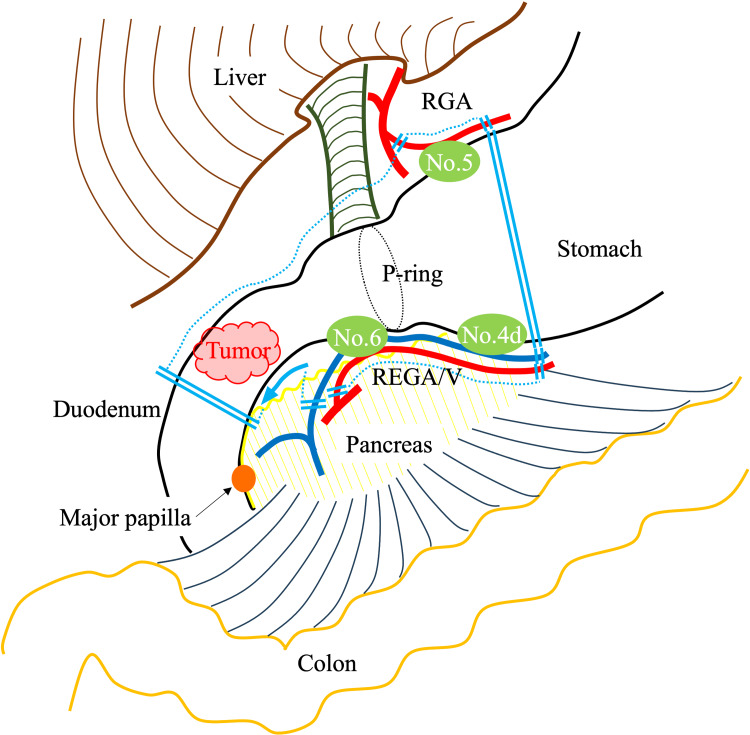
Surgical schema of LSDA Operative schema representing the procedure (based on Case 2). The light blue double lines indicate the resection/transection lines of the stomach and duodenum. The blue dotted lines represent the exact anatomical boundaries of the resected specimen area. The blue arrows indicate the direction and extent of the duodenal mobilization (detachment of the duodenum from the pancreatic head). Numerical labels with "No." (e.g., No. 4d, No. 5, and No. 6) define the regional lymph node stations according to the Japanese Gastric Cancer Treatment Guidelines. The orange oval shape represents the major duodenal papilla. Figure credits: Authors using Microsoft PowerPoint (Microsoft, Redmond, WA, USA). LSDA: laparoscopic segmental duodenectomy with antrectomy; RGA: right gastric artery; RGEA/V: right gastroepiploic artery/vein;  P-ring: pylorus ring.

To ensure adequate oncological margins, we performed intraoperative endoscopy (IOE) to identify the distal border of the tumor. Under IOE guidance, the duodenum was transected onto the distal side of the lesion using a laparoscopic linear stapler (Figure 4c). Subsequently, we dissected lymph node station 5 (suprapyloric node) and divided the right gastric artery. The stomach was then transected into the antrum, including the pylorus. Following retrieval of the resected specimen enclosed in a retrieval bag via a small umbilical incision, the anatomical relationship between the duodenal stump, divided vessels, and fully preserved pancreatic head was clearly confirmed (Figure 4d). 

**Figure 4:**
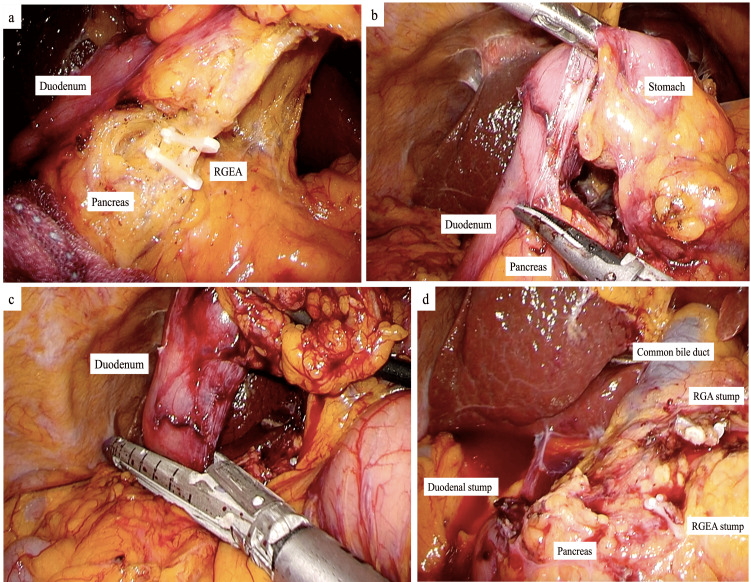
Intraoperative findings (a) Laparoscopic view of RGEA division and station 6 (infrapyloric) lymph node dissection. (b) Duodenal mobilization. The fusion between the duodenum and pancreatic head was carefully dissected. (c) Transection of the duodenum from the anal side of the tumor using a laparoscopic linear stapler under intraoperative endoscopic guidance. (d) Laparoscopic view of the surgical field after specimen retrieval. The duodenal stump, RGEA stump, RGA stump, and preserved pancreatic head are clearly visible. RGA: right gastric artery; RGEA: right gastroepiploic artery.

Regarding the reconstruction method after LSDA, we selected the Billroth II technique rather than the Roux-en-Y approach. Although the Billroth II method carries a potential risk of bile reflux due to the absence of the pylorus, it offers significant advantages in this specific procedure. Specifically, the Billroth II technique is technically simpler, requiring only a single anastomosis. Furthermore, it avoids jejunal transection, thereby minimizing the risks of mesenteric hemorrhage or postoperative internal hernia, complications that are occasionally associated with the Roux-en-Y limb construction. Given the patients' advanced age and frailty, minimizing operative complexity and reducing reconstruction-related complications were prioritized. The operative times were 143 and 162 min, respectively, with minimal blood loss. 

Postoperative course and pathological findings

Both patients had otherwise uneventful postoperative courses. There was no 30- or 90-day mortality, and no reoperation or readmission was required for either patient. Furthermore, no major procedure-specific complications, such as pancreatic fistula, delayed gastric emptying, anastomotic leakage, or symptomatic bile reflux, occurred. Case 1 was discharged on postoperative day 7, while Case 2 was discharged on postoperative day 14 following the successful treatment of a minor catheter-related infection (Clavien-Dindo grade II).

Histopathological examination revealed well-to-moderately differentiated adenocarcinoma (Case 1: pTis N0; Case 2: pT1b N0), with R0 resection achieved in both cases (Figure 5a, 5b). The distal margin length was 15 mm in Case 1 and 7 mm in Case 2. The number of retrieved lymph nodes was 10 in Case 1 and 12 in Case 2, and no metastases were found. Venous and lymphovascular invasion was absent in both cases. Both patients remain recurrence-free at 58 and 35 months, respectively, with preserved nutritional status and quality of life. Notably, neither patient reported clinical symptoms of bile reflux, and follow-up endoscopy revealed no significant reflux gastritis.

**Figure 5:**
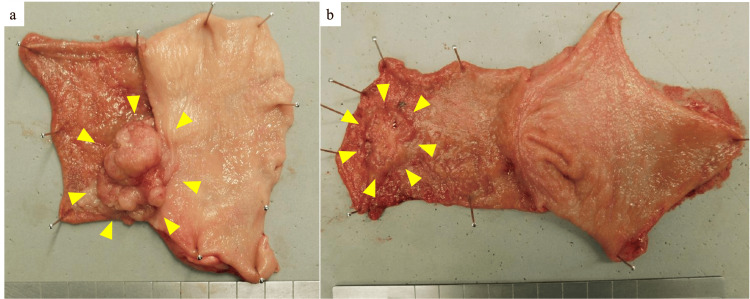
Resected specimens (a) Case 1. Resected specimen showing a localized tumor with sufficient surgical margins. (b) Case 2. Macroscopic findings of the duodenal lesion, which was subsequently confirmed to be a pT1b tumor.

## Discussion

NADC is a rare malignancy, with an annual incidence of 23.7 per 1,000,000 person-years [1]. Although over half of the cases are diagnosed at a localized stage, advanced disease is more common in patients aged ≥80 years, underscoring the need to balance oncological radicality with surgical safety in this population [1].

The treatment strategy for NADC is largely determined by tumor location and depth of invasion. While PD remains the standard curative procedure, its use is limited by substantial postoperative morbidity, particularly in octogenarians. A recent meta-analysis reported higher morbidity and mortality in older patients undergoing PD [6], highlighting the need for less invasive, function-preserving alternatives.

ER is a minimally invasive option for superficial NADC; however, its applicability is limited by the duodenal anatomy, including a thin wall and rich vascularity, which increase the risk of perforation and bleeding. Lesions near the pylorus or superior duodenal angle also pose technical challenges and a risk of inadequate resection margins [9]. These limitations highlight the need for alternative approaches in selected cases.

Current guidelines state that PD remains the standard radical surgery, whereas local resection, including pancreas-preserving partial duodenectomy, may be considered for Stage I tumors to preserve postoperative quality of life [2]. LSDA may provide oncological radicality while minimizing surgical stress in selected patients. Recent reviews report favorable outcomes for limited resections in early-stage NADC, supporting avoidance of PD-related morbidity [10,11].

The 2021 guidelines also recognize LECS as a minimally invasive option [2]. While specific criteria for pure laparoscopic partial duodenectomy are not established, recent studies show that experienced surgeons can safely perform limited resections [12].

Surgical decisions for Case 1 (pTis) and Case 2 (pT1b) were guided primarily by anatomical and functional considerations rather than pathology alone. In Case 1, the 30 mm lesion’s size and proximity to the pylorus made ER high risk due to potential perforation and post-procedural stenosis; LSDA ensured secure margins while preserving gastrointestinal function. In Case 2, suspected deep submucosal invasion required regional lymphadenectomy, which could not be achieved with ER or LECS, making LSDA the optimal approach.

The optimal extent of lymphadenectomy in early-stage NADC remains debated. Duodenal adenocarcinoma shows biological behavior and survival outcomes similar to gastric cancer, highlighting the importance of accurate nodal staging [13]. For tumors in the first portion of the duodenum, the Japanese guidelines identify the infrapyloric (station 6) and posterior pancreatic head (station 13) nodes as sentinel nodes [2]. While station 13 is a potential metastatic site, its dissection requires pancreatic mobilization and carries a high risk of pancreatic fistula. Considering patients’ advanced age, cN0 status, and primary lymphatic drainage toward the pylorus [13], systematic dissection of stations 5 and 6 was performed while omitting station 13 to balance oncological safety with minimal invasiveness.

Recent evidence supports this approach: Miura et al. demonstrated the clinical importance of infrapyloric (station 6) nodes in local control of proximal duodenal cancer [14], and Hisaoka et al. reported that in pT1b non-ampullary duodenal adenocarcinoma, metastases were primarily observed in perigastric nodes, with none in station 13 [15]. Long-term recurrence-free survival (58 and 35 months) further supports the oncological acceptability of this tailored lymphadenectomy strategy in octogenarians.

LSDA offers technical advantages, including procedural standardization and reliability. Localized partial duodenectomy may increase the risk of anastomotic tension or stenosis, whereas extending resection to the gastric antrum provides adequate margins and enables tension-free Billroth II reconstruction. Minimizing surgical stress is especially important in octogenarians, and a recent meta-analysis suggests that Billroth II reconstruction offers shorter operative times with comparable safety when Billroth I is not feasible, facilitating smoother postoperative recovery [16].

Although limited by the small sample size, the long follow-up (up to 58 months) provides strong evidence supporting the long-term oncological safety of LSDA. LSDA appears to be a technically straightforward, oncologically sound alternative to more invasive procedures in selected frail patients.

## Conclusions

LSDA is a technically feasible, function-preserving treatment option for early-stage NADC. It represents a promising approach that can potentially achieve a favorable balance between oncological radicality and minimal invasiveness, particularly in elderly or frail patients with tumors located in the duodenal bulb or superior duodenal angle.
